# Association between peripheral T-Lymphocyte activation and impaired bone mineral density in HIV-infected patients

**DOI:** 10.1186/1479-5876-11-51

**Published:** 2013-02-28

**Authors:** Lidia Gazzola, Giusi Maria Bellistri, Camilla Tincati, Valentina Ierardi, Alessia Savoldi, Angelo Del Sole, Luca Tagliabue, Antonella d’Arminio Monforte, Giulia Marchetti

**Affiliations:** 1 Department of Health Sciences, Clinic of Infectious Diseases, “San Paolo” Hospital, University of Milan, Via A. Di Rudinì, 8, Milan, 20142 Italy; 2 Department of Biomedical Sciences and Technologies and Center of Molecular and Cellular Imaging, “San Paolo” Hospital, University of Milan, Milan, Italy

**Keywords:** Osteopenia, Osteoporosis, Bone mineral density, T-cell activation, Immune senescence

## Abstract

**Background:**

HIV-infected patients display an increased and early incidence of osteopenia/osteoporosis. We investigated whether bone metabolism disorders in HIV-infected patients are related to immune hyperactivation and premature immune senescence.

**Methods:**

Bone mineral density (BMD) was measured by dual-energy X-ray absorptiometry (DXA): low BMD (LBMD) was defined as T-score or z-score < -1. CD4+/CD8+ phenotype (CD38/HLA-DR, CD127, CD28/CD57), and circulating IL-7, TNF-α, RANKL, OPG were measured. The variables with *p* < .05 were evaluated by multivariate logistic regression.

**Results:**

78 patients were enrolled: 55 were LBMD. LBMD patients showed increased activated HDLADR + CD4+ and CD8+ (*p* = .03 and *p* = .002, respectively). Interestingly, no differences in senescent CD28-CD57 + CD4+/CD8+ T-cells were observed between groups. However, LBMD patients displayed a decreased CD4 + CD28- phenotype (*p* = .04) at the advantage of the CD28+ pool (*p* = .03), possibly reflecting heightened apoptosis of highly differentiated CD28-negative cells.

Activated HLADR + CD4+/CD8+ and CD28 + CD4+ cells were independently associated with impaired BMD (AOR = 1.08 for each additional HLADR + CD4+ percentage higher; CI 95%,1.01-1.15; *p* = .02; AOR = 1.07 for each additional HLADR + CD8+ percentage higher; CI 95%,1.01-1.11; *p* = .01; AOR = 1.06 for each additional CD28 + CD4+ percentage higher; CI 95%,1.0-1.13; *p* = .05).

**Conclusions:**

Heightened T-cell activation in HIV-infected patients independently predicts BMD disorders, suggesting a critical role of immune activation in the pathogenesis of osteopenia/osteoporosis, even in patients achieving full viral suppression with HAART.

**Electronic supplementary material:**

The online version of this article (doi:10.1186/1479-5876-11-51) contains supplementary material, which is available to authorized users.

## Introduction

HIV-infected patients are characterized by a high prevalence of osteopenia and osteoporosis [1] that may contribute to an increased risk of pathologic fractures [2].

As clinicians who manage osteopenia/osteoporosis in HIV infection, we should propose screening programs and identify preventive clinical approaches. In this context, a broad comprehension of the pathogenetic mechanisms of bone mineral density (BMD) disorders in HIV/AIDS and a clear definition of the HIV-infected subpopulations who are at an increased risk for osteopenia/osteoporosis are needed to determine the best management of this clinical condition.

Multiple factors are involved in the pathogenesis of altered bone homeostasis in HIV infection [3]. Osteopenia was initially attributed to HAART, especially to regimens that contain protease inhibitors or tenofovir [3]. HIV infection was postulated as a possible cause because HIV-encoded proteins were demonstrated to modulate osteoblast development/function and osteoclast differentiation [4–6].

The elevated incidence of bone co-morbidities in younger HIV-infected individuals compared with the HIV-negative population suggests that these diseases could be the result of premature aging [7]. Similar to other age-related diseases, osteoporosis is initiated and/or worsened by chronic, systemic low-grade inflammatory activity that leads to long-term tissue damage [8].

HIV-infected patients on HAART, despite full virologic control, exhibit a hyperactivated immune profile that is characterized by increased circulating pro-inflammatory cytokines and reduced naïve/central memory T-cell subsets in favor of activated/senescent phenotypes [9–11]. These immunological alterations are similar to those that have been observed in the HIV-uninfected elderly and may contribute to the premature clinical aging of HIV-infected patients, even in the context of virologically suppressive therapy [12–14].

Furthermore, patients with chronic HIV infection display significantly increased plasma levels of interleukin-7 (IL-7), which is a type-1 stromally produced cytokine that has been demonstrated to promote osteoclast activity by upregulating T-cell-derived osteoclastogenic cytokines [15–17]. A substantial reduction in IL-7R-expressing T-cells with impairment of IL-7-mediated signaling has been described [18].

Despite the evidence of a pathogenetic role of T-cell activation and IL-7/IL-7R dysfunction that favors osteoclast formation and bone resorption under inflammatory conditions [19, 20], no studies have specifically addressed the correlations between the HIV-related peripheral immune phenotype and the pathogenesis of bone metabolism disorders.

Therefore, we aimed to evaluate whether HIV-infected patients with impaired BMD are characterized by an activated/senescent immune phenotype and a pro-inflammatory cytokine *milieu* in peripheral blood, which may suggest that there are specific immune pathogenetic pathway(s) that are involved in HIV-related bone disorders. The identification of immunological marker(s) that are associated with impaired BMD could provide additional tools for the early detection of HIV-infected subpopulations who are at an increased clinical risk of osteopenia/osteoporosis and who would greatly benefit from specific screening programs and therapeutic strategies.

## Patients and methods

### Patient enrollment

From June 2006 to July 2008, a cross-sectional study was conducted at the Clinic of Infectious Diseases at “San Paolo” Hospital, University of Milan. All patients provided informed consent. We consecutively enrolled HIV-positive patients of both sexes who were naïve or on stable HAART for at least 12 months and who underwent BMD analysis by dual-energy X-ray absorptiometry (DXA) and PBMC/plasma collection for immunologic analyses. The exclusion criteria were concomitant opportunistic infections and/or primary HIV infection.

### Bone mineral density

BMD at the lumbar spine (LS) and femoral neck (FN) was measured by DXA on a QDR 4500 bone densitometer using Hologic Discovery W. T-scores, which compare subjects with young individuals of the same sex and race, were derived from the National Health and Nutrition Examination Survey (NHANES III). According to WHO criteria, osteopenia was defined by T-scores at the LS and/or FN that were  -1 SD and ≥ -2.5; osteoporosis was defined by T-scores < -2.5 [21]. For pre-menopausal women and men younger than 50 years of age z-score < -1 was used for osteopenia diagnosis, and z-score < -2 for osteoporosis [22, 23].

### Immunophenotypic analysis

All of the immunophenotypic assays were performed on frozen samples. The following fluorochrome-labeled mAbs were used: HLA-DR-FITC, CD38–PE, CD4-PerPC-Cy5.5, and CD8-PerPC-Cy5.5; CD4-Pcy7, CD8-Pcy5, and CD127-PE (BD Biosciences, San Jose, California, USA); and CD57-FITC, CD28-PE, CD8-Pcy5, and CD4-Pcy7 (Beckman Coulter, Hialeah, FL). The following combinations were used: CD4/CD127, CD8/CD127, CD4/CD38/HLA-DR, CD8/CD38/HLA-DR, and CD4/CD8/CD28/CD57. The analysis was conducted using a Cytomics FC500 instrument (Beckman Coulter, Hialeah, FL).

### Quantification of IL-7Rα mRNA by real-time PCR in CD4+ and CD8+ T-cells

Following the isolation of PBMCs from peripheral blood (Ficoll Histopaque, Biochrom AG Seromed, Milan, Italy), CD4+ and CD8+ lymphocytes were separated with magnetic beads (Microbeads, Miltenyi, Bologna, Italy).

Total RNA was isolated from CD4+ and CD8+ T-cells using the commercially available RNeasy Micro Kit (Qiagen, Milan, Italy) according to the manufacturer’s instructions. Potential genomic DNA contamination was removed by incubating the RNA for 15 min at room temperature with RNase-free DNase I (Invitrogen, Carlsbad, CA, USA). From each sample, a variable amount of total RNA was reverse transcribed into cDNA using the SuperScript™ III First-Strand Synthesis System (Invitrogen, Carlsbad, CA, USA) according to the manufacturer’s instructions under the following conditions: 25°C for 10 min, 50°C for 30 min and 85°C for 5 min. After RNase H was added to eliminate potential RNA contamination, a final incubation was conducted at 37°C for 20 min.

IL-7Rα mRNA detection was performed using an Assay-on-Demand Kit (Assay ID Hs00233682_m1, Applied Biosystems, Foster City, CA, USA); the housekeeping gene TFRC (Assay ID Hs99999911_m1, Applied Biosystems) was used as a control as previously described [18].

### Pro-inflammatory and osteoclastogenic cytokines

IL-7 and TNF-α plasma concentrations were evaluated by ELISA (Quantikine HS Human IL-7 and Human TNF-α, R&D Systems, Milan, Italy) according to the manufacturer’s instructions. The plasma concentrations of nuclear factor kappa-B ligand (RANKL) and its soluble receptor antagonist osteoprotegerin (OPG) were measured by ELISA (Human Soluble RANKL Total ELISA, BioVendor, Czech Republic, and Quantikine HS Human OPG, R&D Systems, Milan, Italy) according to the manufacturer’s instructions.

### Statistical analysis

The continuous variables were represented as the median and IQR; the categorical parameters as the absolute numbers and percentage. The differences between LBMD and NBMD patients were assessed by Mann–Whitney test and Pearson’s chi-squared test (continuous parameters), and by Mann–Whitney test and Fisher’s exact test (categorical parameters). Two multivariate logistic regression models were established to identify potential predictors of osteopenia/osteoporosis: we separately analyzed the association between immunological markers on CD4+ T cells (model 1) and CD8+ T cells (model 2) in patients with low BMD. We established two models to assess whether low BMD was independently influenced by the phenotype of circulating CD4+ and CD8+ T-cell subpopulations. Each model was adjusted for demographic and HIV-related variables (age, sex, BMI, hepatitis coinfection, CD4+ count, time on HAART). All of the statistical analyses were performed using SPSS software (version 18.01, SPSS). All of the tests were 2-sided, and *p*≤0.05 was considered to be a cut-off for statistical significance.

## Results

### Demographics, HIV-related parameters and bone mineral density of the study population

A total of 78 HIV-infected patients were recruited. The patient demographics are represented in Table 1. Regarding HIV infection, patients were characterized by a mean CD4+ count of 507 cell/mm^3^; most of the patients were on stable HAART, with undetectable viremia and a mean time of 6.7 years of HAART exposure. In addition, the enrolled patients exhibited a low prevalence of the common risk factors for osteoporosis.

**Table 1:** **Characteristics of the study population and analysis of the demographic characteristics and HIV-related parameters between low bone mineral density (LBMD) patients (T-score < -1) and normal bone mineral density (NBMD) patients (T-score ≥ -1)**

	TOTAL (n = 78)	LBMD (n = 55)	NBMD (n = 23)	P^§^
**Demographic characteristics**				
male	68%	66.7%	69.6%	0.80*
mean age (IC 95%)	46 (43–48)	46 (43–48)	45 (40–50)	0.81
mean BMI (IC 95%)	23.4 (22.5-24.3)	23.4 (22.5-24.9)	24.6 (23.0-26.2)	0.16
**HIV-related parameters**				
AIDS diagnosis	22%	23%	17%	0.54*
time from HIV diagnosis (IC 95%)	7.8 years (6.3-9.3)	7.8 years (5.8-9.8)	7.8 years (5.5-10.4)	0.99
mean CD4 nadir (IC 95%)	268 cell/mm^3^ (226–310)	266 cell/mm^3^ (214–317)	265 cell/mm^3^ (195–336)	0.98
mean current CD4 (IC 95%)	507 cell/mm^3^ (437–577)	490 cell/mm^3^ (400–579)	512 cell/mm^3^ (407–617)	0.77
mean CD4/CD8 ratio (IC 95%)	0.7 (0.59-0.80)	0.7 (0.54-0.77)	0.8 (0.56-1.02)	0.25
co-infection with viral hepatitis				
HCV Ab positivity	10%	12%	4%	0.26**
HBsAg positivity	5%	3%	8%	0.63**
naïve patients	26%	28%	17%	0.31**
mean time of HAART exposure (IC 95%)	4.6 years (3.4-5.9)	4.0 years (2.6-5.5)	6.2 years (3.8-8.7)	0.13
type of HAART				0.40*
TDF-based backbone	53%	54%	53%	
PI-based	33%	34%	35%	
NNRTI-based	37%	33%	48%	
undetectable viremia (HIV-RNA <40 UI/mL) (in patients on HAART)	92%	88%	94%	1.00*

^**§**^p, comparison between LBMD and NBMD patients by *Pearson’s chi-squared test and **Fisher’s exact test.

DXA analysis was performed on all 78 of the patients who were enrolled in the study according to the inclusion criteria: 23/78 patients (30%) exhibited a BMD within a normal range at both sites, 47/78 patients (60%) had a reduction in BMD that indicated osteopenia and 8/78 patients (10%) had a BMD reduction that indicated osteoporosis.

Patients who were characterized by a low BMD, including both osteopenic and osteoporotic patients (LBMD: defined by a T-score or z-score < -1), were comparable to normal BMD patients (NBMD: defined by a T-score or z-score ≥ -1) according to the demographic characteristics and common HIV-related parameters, such as the CD4+ nadir and current value, time from HIV diagnosis, HAART exposure and type of HAART, as represented in Table 1.

### HIV-positive patients with low BMD display a hyperactivated peripheral T-cell phenotype

**Figure 1:**
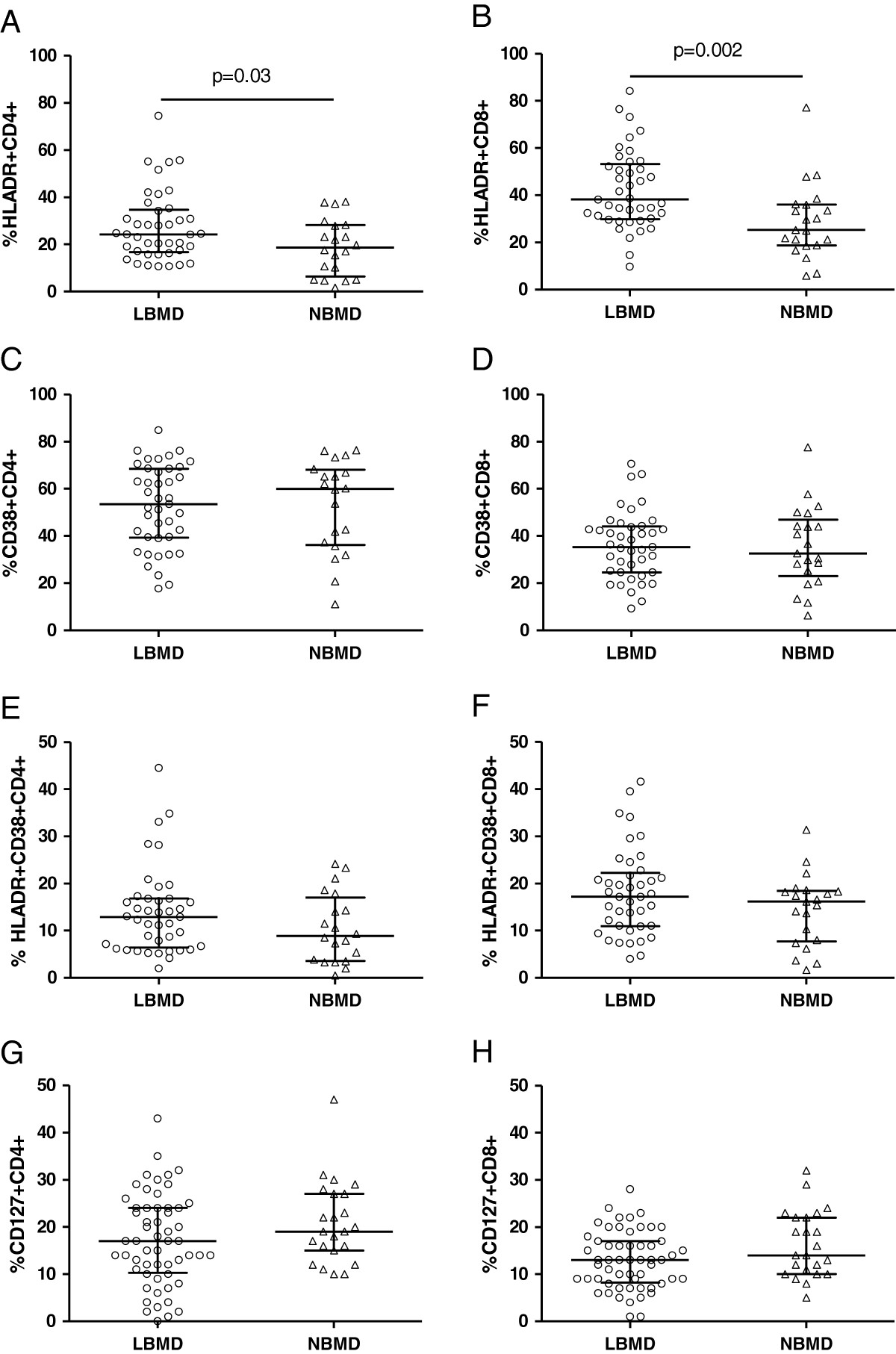
**An immunophenotypic analysis of peripheral blood T-cell activation was performed on sixty-two patients (41 LBMD patients and 21 NBMD patients).** Patients with LBMD are shown in the circles, and patients with NBMD are shown in the triangles. The median percentage of HLADR + on CD4+ (**A**) and CD8+ (**B**) T-cells. The median percentage of CD38+ on CD4+ (**C**) and CD8+ (**D**) T-cells. The median percentage of HLADR + CD38+ on CD4+ (**E**) and CD8+ (**F**) T-cells. The bars correspond to the IQR. The expression of CD127 on CD4+ and CD8+ T-cells was evaluated for all seventy-eight patients. The median percentage of CD127 + CD4+ (**G**) and CD8+ (**H**) T-cells in PBMCs. The bars correspond to the IQR.

We compared CD4+ and CD8+ T-cell activation in a subgroup of 62 patients for whom viable PBMCs were available (21 NBMD patients and 41 LBMD patients) (Figure 1).

According to the analysis of HLADR expression, patients with LBMD demonstrated an increased frequency of activated CD4+ T-cells (HLADR + CD4+ 24.3% [IQR = 16.7%-34.7%] in LBMD patients and 18.6% [IQR = 6.3%-28.2%] in NBMD patients; *p* = .03) and CD8+ T-cells (HLADR + CD8+ 38.2% [IQR = 29.8%-53.2%] in LBMD patients and 25.3% [IQR = 18.75%-36.15%] in NBMD patients, *p* = .002) (Figures 1A and B).

No differences in the expression of CD38 on CD4+ and CD8+ T-cells were observed between the LBMD and NBMD patients (CD38 + CD4+ 53.5% [IQR = 39.2%-68.55%] in LBMD patients and 59.9% [IQR = 36.2%-68.0%] in NBMD patients, *p* = .90; CD38 + CD8+ 35.3% [IQR = 24.6%-44.0%] in LBMD patients and 32.6% [IQR = 23.0%-46.9%] in NBMD patients, *p* = .92) (Figures 1C and D). Similarly, no significant differences were observed in the co-expression of CD38 and HLADR on CD4+ and CD8+ T-cells among the LBMD and NBMD patients (HLADR + CD38 + CD4+ 12.9% [IQR = 6.4%-16.8%] in LBMD patients and 8.9% [IQR = 3.6%-17%] in NBMD patients, *p* = .16; HLADR + CD38 + CD8+ 17.2% [IQR = 10.9%-22.2%] in LBMD patients and 16.2% [IQR = 7.7%-18.4%] in NBMD patients, *p* = .17) (Figures 1E and F).

We observed an association between LBMD and increased CD4+ and CD8+ T-cell activation; therefore, we investigated whether a similar finding could be obtained in the subgroup of patients who were virologically suppressed under HAART. In 57/78 patients who were on stable virologically suppressive HAART (HIV RNA < 50 cp/ml), the patients with LBMD displayed increased CD8+ T-cell activation (HLADR + CD8+ 36.6% [IQR = 30.7%-51.6%] in LBMD patients and 29.6% [IQR = 17.7%-36.1%] in NBMD patients, *p* = .008) but no significant differences in CD4+ T-cell activation (HLADR + CD4+ 23.1% [IQR = 16.7%-36.4%] in LBMD patients and 19.7% [IQR = 10.4%-28.1%] in NBMD patients; *p* = .08).

### HIV-positive patients with low BMD exhibit a contraction of the central memory CD127 + CD8+ T-cell pool

The loss of IL-7Rα (CD127) expression on CD8+ T-cells defines a population at a terminal state of differentiation and has been associated with a higher level of T-cell activation in the course of HIV infection [24]; therefore, we compared CD127 expression on the peripheral T-cells of LBMD patients *vs.* NBMD patients. The analysis were performed in 78 patients, no differences in the proportion of CD4+ T-cells that expressed CD127 were observed between LBMD and NBMD patients (CD127 + CD4+ T-cells 17% [IQR = 10.2%-24%] and 19% [IQR = 15%-27%], respectively, *p* = .16) (Figure 1G). Interestingly, patients with LBMD demonstrated a non significant trend toward a reduced frequency of CD8+ T-cells that expressed CD127 compared with NBMD patients: the proportion of CD127 + CD8+ T-cells was 13% (IQR = 8.2%-17%) in LBMD patients and 14% (IQR = 10%-22%) in NBMD patients (*p* = .06) (Figure 1H).

After observing a trend toward a lower percentage of CD127 + CD8+ T-cells in patients with LBMD, we investigated whether the decreased expression of CD127 on the CD8+ T-cell surface corresponded to a reduced production of CD127. We measured IL-7Rα mRNA in CD8+ T-cells from a subgroup of 22 unselected patients (7 NBMD patients and 15 LBMD patients). The patients who were affected by LBMD demonstrated IL-7Rα mRNA levels in CD8+ T-cells that were comparable to patients with NBMD (median copies of IL-7Rα mRNA/100 ng TFRC: 753 [IQR = 207-1,014] in LBMD patients and 1,037 [IQR = 581-3,615] in NBMD patients, *p* = .14).

### HIV-positive patients with low BMD display a peripheral cytokine *milieu* comparable to patients with normal BMD

After observing a hyperactivated T-cell phenotype in LBMD patients, we evaluated whether these patients were characterized by a pro-inflammatory and osteoclastogenic cytokine pattern in peripheral blood (TNF-α, IL-7, RANKL, and OPG). Analysis were performed on a subgroup of 42 patients (14 NBMD and 28 LBMD), LBMD patients displayed no differences in median TNF-α plasma levels compared with NBMD patients (4.9 pg/mL [IQR = 1.7-15.8] and 2.4 pg/mL [IQR = 1.6-16.4], respectively, *p* = .48) or in median IL-7 plasma levels (6.5 pg/mL [IQR = 2.4-13.4] in LBMD patients and 8.0 pg/mL [IQR = 3.0-9.7] in NBMD patients, *p* = .91).

Similarly, the analysis of osteoclastogenic cytokines showed no differences in median RANKL plasma levels between LBMD and NBMD patients (5,872 pg/mL [IQR = 2,823-10,259] in LBMD patients and 8,438 pg/mL [IQR = 6,421-11,766] in NBMD patients, *p* = .08) or in median plasma OPG levels (550 pg/mL [IQR = 420-1,001] in LBMD patients and 860 pg/mL [IQR = 390-1,197] in NBMD patients, *p* = .97).

### Analysis of peripheral immune-senescent CD4+ and CD8+ T-cell patterns

Based on the phenotype data that indicated high activation in LBMD patients, we investigated the immune senescent pattern of CD4+ and CD8+ T-cells in LBMD patients and NBMD patients according to the expression of CD28 and CD57. T-cell immune senescence was analyzed in the same subgroup of 62 patients for whom T-cell activation data were available.

**Figure 2:**
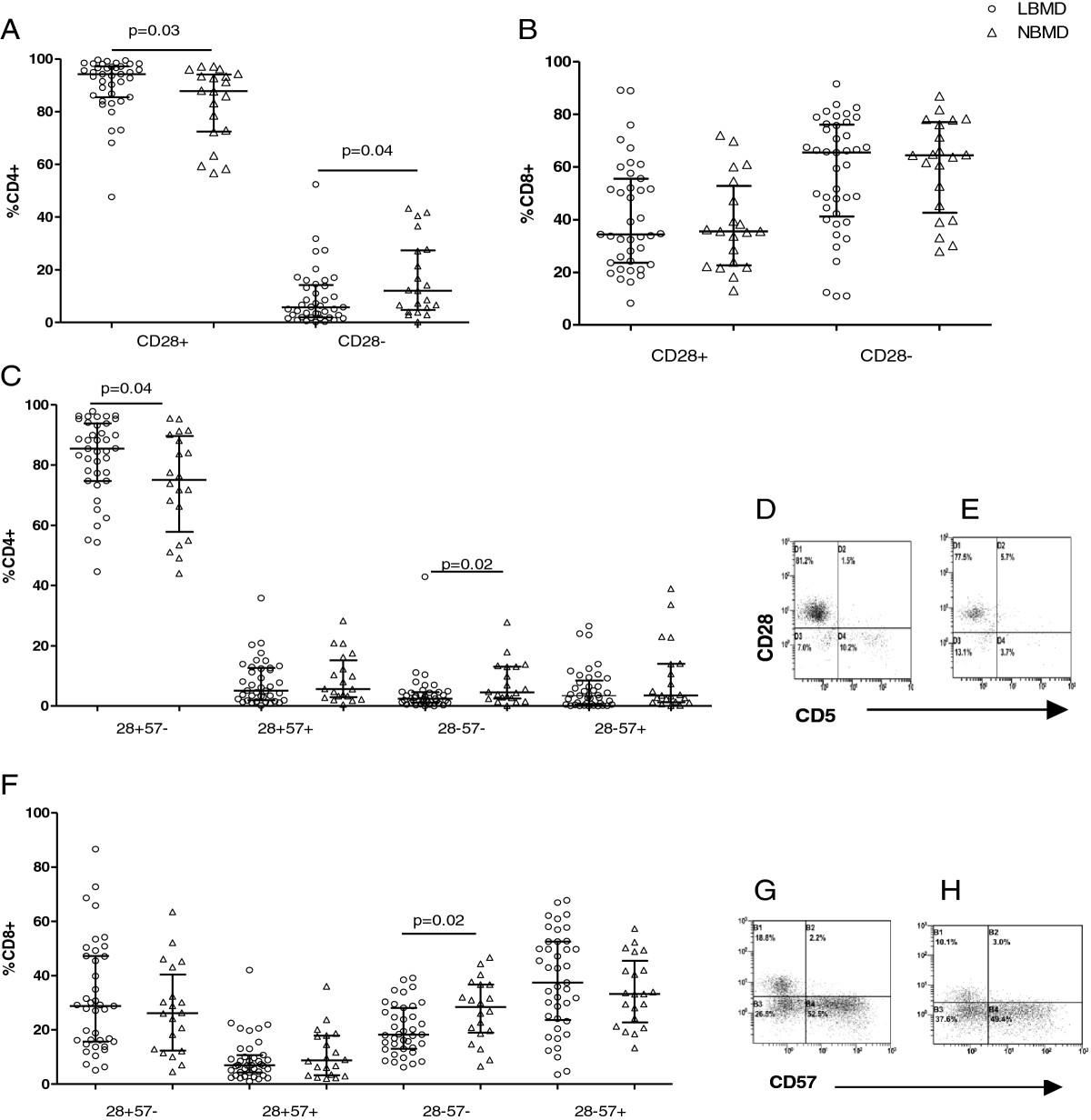
**The immune senescent pattern of CD4+ and CD8+ T-cells.** Patients with LBMD are shown in the circles, and patients with NBMD are shown in the triangles. The bars correspond to the IQR. **A**) The median percentage of CD28+ and CD28-CD4+ T-cells. **B**) The median percentage of CD28+ and CD28-CD8+ T-cells. **C**) The median percentage of CD4+ T-cell sub-populations as defined by CD28 and CD57 co-expression (CD28 + CD57-, CD28 + CD57+, CD28-CD57-, and CD28-CD57+). **D**) and **E**) are representative plots that show the senescence markers (CD57 and CD28) on the CD4+ T-cells of patients with LBMD (**D**) and patients with NBMD (**E**). The data were compensated and analyzed using CXP 2.2 software (Beckman-Coulter). **F**) The median percentage of CD8+ T-cell sub-populations as defined by CD28 and CD57 co-expression (CD28 + CD57-, CD28 + CD57+, CD28-CD57-, and CD28-CD57+). **G**) and **H**) are representative plots that show the senescence markers (CD57 and CD28) on the CD8+ T-cells of patients with LBMD (**G**) and patients with NBMD (**H**). The data were compensated and analyzed using CXP 2.2 software (Beckman-Coulter).

Unexpectedly, we observed a higher frequency of CD28 + CD4+ T-cells in LBMD patients compared with NBMD patients (94.2% [IQR = 85.5%-97.1%] in LBMD patients and 87.8% [IQR = 72.4%-94.1%] in NBMD patients, *p* = .03) and a lower frequency of antigen-experienced CD28-CD4+ T-cells in LBMD patients compared with NBMD patients (5.8% [IQR = 1.9%-14.2%] in LBMD patients and 12.1% [IQR = 4.8%-27.4%] in NBMD patients, *p* = .04) (Figure 2A). No differences in CD28 expression on CD8+ T-cells were observed between the patient groups (CD28 + CD8+ 34.3% [IQR = 23.6%-55.6%] in LBMD patients and 35.5% [IQR = 22.6%-52.8%] in NBMD patients, *p* = .92; CD28-CD8+ T-cells 65.5% [IQR = 22.6%-55.6%] in LBMD patients and 64.4% [IQR = 42.6%-77%] in NBMD patients, *p* = .89) (Figure 2B).

The analysis of CD28 and CD57 co-expression on T-lymphocytes resulted in different phenotypes according to bone mineral density (Figure 2). There were no differences in the frequencies of senescent CD28-CD57 + CD4+ T-cells (3.4% [IQR = 0.5%-8.5%] in LBMD patients and 3.5% [IQR = 1.3%-14%] in NBMD patients, *p* = .24) (Figure 2C); however, LBMD patients displayed higher CD28 + CD57-CD4+ T-cells (85.4% [IQR = 74.7%-93.8%] in LBMD patients and 75.0% [IQR = 57.8%-89.6%] in NBMD patients, *p* = .04) and lower CD28-57-CD4+ T-cells (2.4% [IQR = 1.1%-4.6%] in LBMD patients and 4.5% [IQR = 2.5%-13.1%] in NBMD patients, *p* = .02) (Figure 2C). No differences in CD28 + CD57 + CD4+ T-cells were observed (5.1% [IQR = 1.9%-12.6%] in LBMD patients and 5.6% [IQR = 2.9%-15.2%] in NBMD patients, *p* = .56) (Figure 2C).

Similar CD28/CD57-expressing profiles were observed with CD8+ T-cells. LBMD patients exhibited a lower frequency of CD28-CD57-CD8+ T-cells (18.2% [IQR = 13%-28.1%] in LBMD patients and 28.4% [IQR = 18.9%-36.7%] in NBMD patients, *p* = .02), whereas no differences in any other subpopulations were observed (CD28 + CD57-CD8+ 28.8% [IQR = 15.7%-47.2%] in LBMD patients and 26.1% [IQR = 12.4%-40.4%] in NBMD patients, *p* = .42; CD28 + CD57 + CD8+ 7.0% [IQR = 4.3%-10.7%] in LBMD patients and 8.7% [IQR = 3.3%-17.8%] in NBMD patients, *p* = .49; CD28-CD57+ 37.4% [IQR = 23.6%-52.5%] in LBMD patients and 33.2% [IQR = 22.8%-45.4%] in NBMD patients, *p* = .33) (Figure 2F).

### CD4+ and CD8+ T-cell activation is a risk factor that is independently associated with low BMD

A hyperactivated immune pattern is associated with low BMD; therefore, we investigated the predictive role of immunological parameters after controlling for potentially confounding demographic and HIV-related parameters (Table 2). Interestingly, CD4+ and CD8+ T-cell activation was independently associated with low BMD (for each point increase in percentage of HLADR + CD4+: AOR = 1.08, CI 95%, 1.01-1.15, *p* = .02 and for each point increase in percentage of HLADR + CD8+: AOR = 1.06; CI 95%, 1.01-1.11, *p* = .01). The expression of CD28 on CD4+ T-cells was associated with low BMD at a limit of statistical significance (for each point increase in percentage of CD28 + CD4+: AOR = 1.07; CI 95%, 1.00-1.13, *p* = .04).

**Table 2:** **Predictors of osteopenia/osteoporosis**

	Model 1*			Model 2^		
	AOR	CI 95%	*p*	AOR	CI 95%	*p*
sex (male)	1.11	0.24-5.17	.9	0.90	0.21-3.82	.9
age (for each additional year)	1.03	0.97-1.10	.3	1.01	0.95-1.07	.7
BMI (for each additional point)	1.00	0.99-1.00	.9	1.00	0.99-1.00	.8
hepatitis co-infection	1.39	0.15-12.7	.8	1.84	0.25-13.4	.5
CD4+ nadir (for 50 cells or more)	1.08	0.88-1.32	.4	1.04	0.87-1.24	.7
time on HAART (for each additional year)	0.92	0.80-1.05	.2	0.93	0.81-1.07	.3
**HLA-DR + CD4+ (for each additional %)**	**1.08**	**1.01-1.15**	**.02**			
**CD28 + CD4+ (for each additional %)**	**1.06**	**1.0-1.13**	**.04**			
**HLA-DR + CD8+ (for each additional %)**				**1.06**	**1.01-1.11**	**.01**
CD127 + CD8+ (for each additional %)				0.94	0.83-1.06	.3

*Model 1: a multivariate logistic regression was performed to explore the association between CD4+ activation and senescence with low BMD, adjusting for demographic and HIV-related parameters. ^Model 2: a multivariate logistic regression was performed to explore the association between activated and central memory CD8+ T cells with low BMD after correction for demographic and HIV-related parameters.

As shown in Table 2, no other variables were independently associated with pathologic DXA.

## Discussion

The pathogenesis of bone metabolism disorders in HIV-infected patients has been attributed to either HIV or several antiretroviral drugs, such as tenofovir or protease inhibitors [4–6, 25]. Recently, a pathogenetic model attributed the early onset of osteopenia/osteoporosis to the generalized inflammation and immunosenescence that characterizes HIV-infected populations, including in the context of virologically suppressive HAART [12, 13].

In this study, we investigated whether HIV-infected patients who were affected by osteopenia/osteoporosis displayed a circulating T-cell immune phenotype that indicated hyperactivation and premature senescence.

In HIV-infected patients who were affected by low BMD, we demonstrated the following: 1) higher peripheral T-cell activation, 2) less pro apoptotic CD28-negative CD4+ T-cells despite no changes in senescent CD28-57+ T-cells, and 3) a highly activated T-cell phenotype as an independent predictor of impaired BMD.

Elevated immune activation is the main pathogenetic mechanism that is involved in HIV infection with and without HAART: it is predictive of the clinical progression to AIDS [24] and has recently been demonstrated to be associated with the emergence of co-morbidities, such as cardiovascular disease and intima thickness [26, 27].

For the first time, our study demonstrates the involvement of HIV-related immune activation in the pathogenesis of bone co-morbidities. The immune phenotype in the peripheral blood of patients with pathological BMD is characterized by a higher frequency of activated T-cells compared with patients with normal BMD. In addition, the association between T-cell activation and low BMD was confirmed in patients on stable HAART with full virological suppression. Interestingly, in the multivariate logistic regression analysis that was adjusted for demographics and HIV- and HAART-related parameters, a higher frequency of activated CD4+/CD8+ T-cells was an independent predictor of osteopenia/osteoporosis.

Similarly, we observed that the immune phenotype in the peripheral blood of patients with impaired BMD was characterized by a non significant trend toward lower CD127-expressing central memory CD8+ T-cells compared with patients with normal BMD.

In HIV/AIDS, the expansion of CD8+ T-cells that lack CD127 has been consistently described [28–31] as a functional status of terminal differentiation [32, 33] and has been attributed to chronic antigenic stimulation.

Thus, higher T-cell activation with contraction in the compartment of central memory CD127 + CD8+ T-cells in patients who are affected by osteopenia/osteoporosis may suggest increased T-cell turnover with skewed maturation of CD8+ T-cells.

Osteoporosis is not typically considered to be an immune-mediated disorder; however, recent data have indicated an overlapping pathway between bone physiology and the biology of inflammation that involves the T-lymphocyte compartment. Activated T-cells affect bone physiology by producing RANKL and pro-inflammatory cytokines (e.g., IL-1 and TNF-α), which promote osteoclast activity [34] and stimulate stromal cells to produce osteoclastogenic IL-7 [15].

Thus, after observing a hyperactivated circulating T-cell immune profile in patients with bone metabolism disorders, we investigated the pro-inflammatory osteoclastogenic cytokine *milieu*.

Despite a non significant trend toward a higher level of TNF-α in patients with low BMD, the levels of IL-7 and osteoclastogenic cytokines were comparable among LBMD and NBMD subjects. More than 70% of the patients were on long-term HAART; therefore, this finding is in agreement with recent data that demonstrated an increase in the osteoclastogenic cytokines RANKL and TNF-α within the first weeks of therapy with no additional surges [35]. Moreover, the DXA analysis provides a static picture of BMD; however, the cytokine *milieu* depicts the current balance of bone remodeling processes and may be influenced by many factors, mainly the effect of protease inhibitors [36].

We next determined whether these LBMD patients were characterized by an immune senescent pattern in peripheral blood.

Despite increased activation, no differences in the proportion of senescent (CD28-CD57+) CD4+ and CD8+ T-cells were observed in patients with impaired BMD compared with patients with normal BMD.

Unexpectedly, patients with impaired BMD displayed a circulating CD4+ T-cell immune phenotype that was enriched for CD28-expressing cells at the disadvantage of the highly differentiated CD28-negative pool. Interestingly, this CD4+ immune phenotype was independently associated with osteopenia/osteoporosis in the multivariate logistic regression analysis.

The downregulation of CD28 has been extensively described in CD8+ T-cells as a hallmark of chronic antigenic stimulation, even in the course of HIV infection. With each repetitive stimulation/proliferation round, CD28 expression is irreversibly downregulated on the CD8+ surface, leading to the accumulation of antigen-experienced (CD28-negative) CD8+ T-cells with shortened telomeres, impaired proliferative ability and decreased susceptibility to apoptosis.

In addition, the loss of CD28 is observed in CD4+ T-cells during chronic immune activation but at a significantly lower rate than CD8+ [37]. Despite the evidence of CD28-CD8+ T-cell accumulation in HIV infection, there are no data on CD28-CD4+ T-cell accumulation.

In our study, patients who were affected by osteopenia/osteoporosis displayed high levels of T-cell activation and a reduction in the CD28-CD4+ T-cell pool. As a possible explanation for this finding, Vivar *et al.* recently demonstrated that CD28-negative T cells, although senescent, display a proapoptotic phenotype, as suggested by high Fas and decreased IL7Rα and Bcl-2 expression [38]. These T-cells are highly susceptible to apoptosis, which results in increased apoptosis during HIV replication and generalized immune activation. Therefore, the contraction of the CD28-negative CD4+ T-cell pool that we observed in patients with bone disorders may have resulted from increased activation-induced apoptosis.

The main limitation to this study is the population that was enrolled, which included both HIV-infected patients who were naïve to antiretroviral therapy and patients undergoing HAART. Many factors may have played a role in the pathogenesis of osteoporosis in these two distinct populations because several antiretroviral medications may directly decrease bone mineral density and act as confounding factors.

## Conclusion

In conclusion, to our knowledge, this is the first study to provide evidence of an association between osteopenia/osteoporosis and heightened T-cell activation in HIV-infected patients. These results may have important clinical implications in research focused on the assessment of subpopulations of HIV-infected patients who are at increased risk of osteopenia/osteoporosis. Moreover, our data highlight the role of immune activation in the pathogenesis of HIV-related bone metabolism disorders and support the need for additional longitudinal studies to investigate the role of biomarkers of immune activation as predictors of noninfectious co-morbidities.

## Authors’ original submitted files for images

Below are the links to the authors’ original submitted files for images. Authors’ original file for figure 1 Authors’ original file for figure 2
