# Supplementary figures and images for: Association between peripheral T-Lymphocyte activation and impaired bone mineral density in HIV-infected patients
Source: J Transl Med. 2013 Feb 28;11:51. doi: 10.1186/1479-5876-11-51 (PMC3598927; doi:10.1186/1479-5876-11-51)

**A**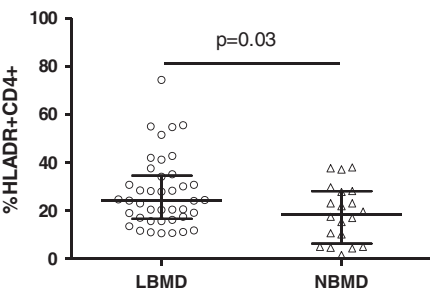**B**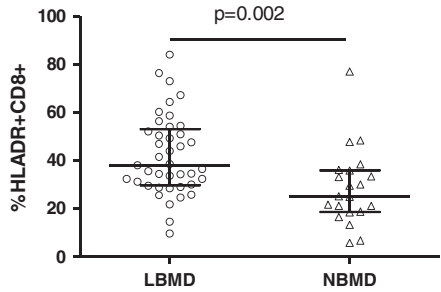**C**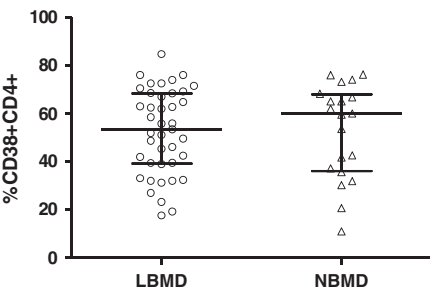**D**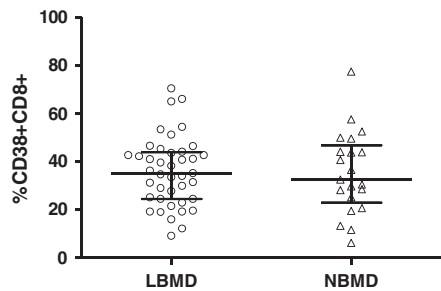**E**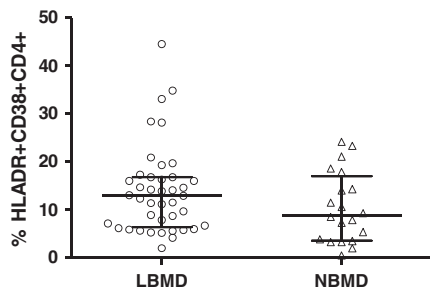**F**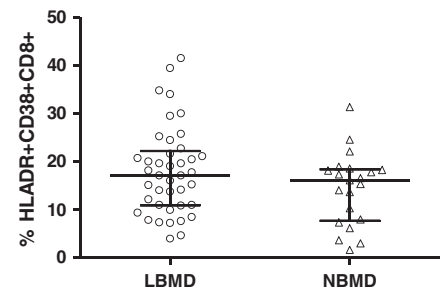**G**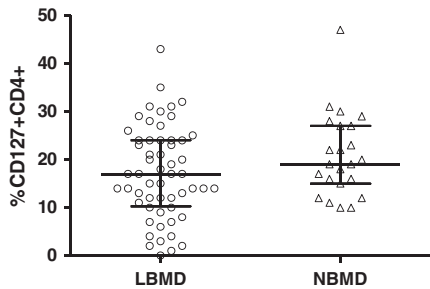**H**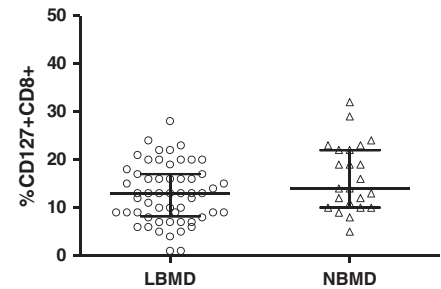

Supplement: Supplementary file 1 — Authors’ original file for figure 1 [file 12967_2012_1434_MOESM1_ESM.pdf]

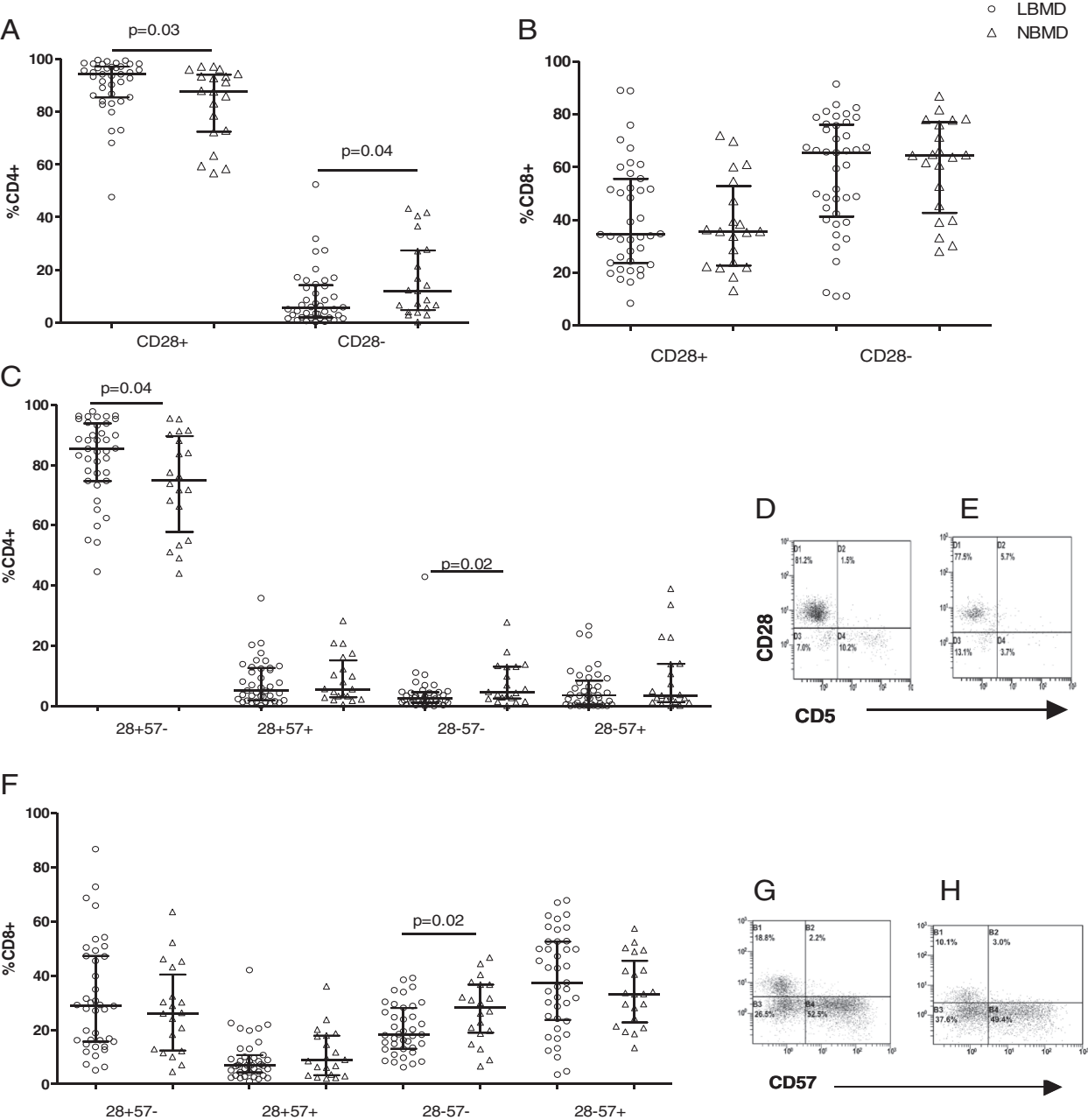

Supplement: Supplementary file 2 — Authors’ original file for figure 2 [file 12967_2012_1434_MOESM2_ESM.pdf]
